# GM-CSF driven myeloid cells in adipose tissue link weight gain and insulin resistance via formation of 2-aminoadipate

**DOI:** 10.1038/s41598-018-29250-8

**Published:** 2018-07-31

**Authors:** Deanna L. Plubell, Alexandra M. Fenton, Phillip A. Wilmarth, Paige Bergstrom, Yuqi Zhao, Jessica Minnier, Jay W. Heinecke, Xia Yang, Nathalie Pamir

**Affiliations:** 1 Department of Medicine, Knight Cardiovascular Institute, Oregon Health & Science University, Portland, OR USA; 2 Proteomics Shared Resource, Oregon Health & Science University, Portland, OR USA; 3 Department of Integrative Biology and Physiology, University of California, Los Angeles, CA USA; 4 Department of Medicine, University of Washington, Seattle, WA USA

## Abstract

In a GM-CSF driven myeloid cell deficient mouse model (*Csf2*^−/−^) that has preserved insulin sensitivity despite increased adiposity, we used unbiased three-dimensional integration of proteome profiles, metabolic profiles, and gene regulatory networks to understand adipose tissue proteome-wide changes and their metabolic implications. Multi-dimensional liquid chromatography mass spectrometry and extended multiplex mass labeling was used to analyze proteomes of epididymal adipose tissues isolated from *Csf2*^+/+^ and *Csf2*^−/−^ mice that were fed low fat, high fat, or high fat plus cholesterol diets for 8 weeks. The metabolic health (as measured by body weight, adiposity, plasma fasting glucose, insulin, triglycerides, phospholipids, total cholesterol levels, and glucose and insulin tolerance tests) deteriorated with diet for both genotypes, while mice lacking Csf2 were protected from insulin resistance. Regardless of diet, 30 mostly mitochondrial, branch chain amino acids (BCAA), and lysine metabolism proteins were altered between *Csf2*^−/−^ and *Csf2*^+/+^ mice (FDR < 0.05). Lack of GM-CSF driven myeloid cells lead to reduced adipose tissue 2-oxoglutarate dehydrogenase complex (DHTKD1) levels and subsequent increase in plasma 2-aminoadipate (2-AA) levels, both of which are reported to correlate with insulin resistance. Tissue DHTKD1 levels were >4-fold upregulated and plasma 2-AA levels were >2 fold reduced in *Csf2*^−/−^ mice (p < 0.05). GM-CSF driven myeloid cells link peripheral insulin sensitivity to adiposity via lysine metabolism involving DHTKD1/2-AA axis in a diet independent manner.

## Introduction

Diets high in fat and cholesterol accelerate expansion of adipose tissue (obesity) and lead to structural and molecular changes within adipose tissue accompanied with an array of metabolic consequences such as insulin resistance, diabetes and cardiovascular disease^1^.

Adipose tissue dendritic cells have been shown to be independent contributors to obesity related insulin resistance^2^. Dendritic cell maturation is stimulated by granulocyte macrophage–colony stimulating factor (GM-CSF), which is produced by various cell types^3–5^. *In vivo*, dendritic cells express the hematopoietic marker CD45 and the integrin CD11C, and they constitutively express major histocompatibility complex class II (MHCII)^6^. *In vitro* GM-CSF prompts bone marrow cells to generate bone marrow-derived dendritic cells (BM-DCs)^7,8^. GM-CSF-induced BM-DCs express CD11B, CD11C, and MHCII at higher levels than bone marrow-derived macrophages (also found in white adipose tissue), both at the plasma membrane^9^ and the mRNA level^10^. Furthermore, mice lacking GM-CSF (*Csf2*^−/−^ mice) have fewer CD11C^+^ cells in their atherosclerotic plaques and white adipose tissue of lean mice indicating that GM-CSF modulates tissue levels of CD11C^+^ cells *in vivo* without major changes in other myeloid populations^11,12^. These mice display improved peripheral insulin sensitivity in spite of increased adiposity by reducing inflammation in adipose tissue^12,13^.

White adipose tissue contains the stromal vascular fraction (SVF), which includes vascular endothelial cells, fibroblasts, and several types of immune cells^14^. The adipose tissue metabolic response to diet induced obesity is often studied by isolation of the stromal vascular fraction by focusing on monocyte derived myeloid cells^15^. The methods to assess global adipose tissue response to diet induced obesity are currently inadequate. The last two decades have seen continuous improvements in “omics” approaches that redefined the way we explore the relationships between genetics, molecular pathways, and disease phenotypes. Recent advances in adipose tissue proteomics^16,17^ now enable an additional molecular layer which can be coupled with genomic, metabolomics, and phenotypic information using systems biology approaches in complex multifactorial diseases such as obesity and insulin resistance.

The majority of obese individuals develop insulin resistance and type 2 diabetes; however, approximately 10–25% of these individuals seem to remain insulin sensitive and metabolically ‘healthy’^18^. Increased adipose tissue serves as an important pathogenic site in the development of type 2 diabetes^19^, and the prevalence of metabolically healthy obesity has been attributed to a normal adipose tissue function^1^. Mice lacking GM-CSF have increased adiposity in both lean^12^ and obese states^13^ despite favorable insulin sensitivity. Therefore, they provide a model to study the biochemical mechanisms that underlie the functional properties of “healthy” adipose tissue and can help explain the difference between obese individuals with normal glucose tolerance and those with type 2 diabetes.

Through unbiased three-dimensional integration of proteome profiles, metabolic profiles, and gene regulatory networks, we have identified unique sets of proteins and gene networks that are modulated by GM-CSF driven myeloid cells and that characterize the changes within adipose tissue in response to high fat or high fat plus cholesterol feeding. Most interestingly, the lack of GM-CSF driven myeloid cells leads to decreased fatty acid metabolism and increased amino acid metabolism regardless of diets. DHTKD1 (2-oxoglutarate dehydrogenase complex) catalyzes the overall conversion of 2-oxoglutarate to succinyl-CoA and CO_2_, and impacts plasma 2-aminoadipate (2-AA) levels, which are elevated in diabetic state^20^. Agreeably, in our study, lysine metabolism was highlighted by robust increase in DHTKD1 levels that are associated with lower plasma 2-AA levels in *Csf2*^−/−^ mice. Our studies reveal a divergent role for adipose tissue GM-CSF driven myeloid cells: While they protect from adiposity they also participate in diet-independent insulin sensitivity via the lysine metabolism involving the Dhtkd1/2-AA axis.

## Materials and Methods

### Mice

All studies were approved by the Institutional Animal Care and Use Committee of the University of Washington. All experiments were performed in accordance with relevant guidelines and regulations. Diets and tissue collection is detailed in Supplemental Material.

### Metabolic measurements

Insulin and glucose tolerance tests were performed after a 4-h fast. Mice were injected intraperitoneally with human insulin (1.0 U/kg body weight; Eli Lilly, Indianapolis, IN) or glucose (1 mg/g body weight) (20) and blood glucose levels were measured at baseline, 15-, 30-, 60-, and 120-minute time points. At the time of sacrifice, blood was collected (post 4-h fast) for insulin, triglyceride, phospholipid, and cholesterol measurements using an ultra-sensitive insulin ELISA (EMD Millipore, Billerica, MA), an L-Type TG M assay (Wako Diagnostics, Richmond, VA), a Phospholipids C assay (Wako Diagnostics, VA), and an Amplex Red Cholesterol Assay Kit (Invitrogen, CA), respectively.

### Adipose tissue fractionation, flow cytometry, and cell quantification

The detailed protocols of this study are available in the Supplemental Material. Under sterile conditions, epididymal adipose tissue was extracted and separated into stromal vascular cell and adipocyte fractions as previously described^21^ and Supplemental Material. After isolation of stromal vascular fraction (SVF) from the epididymal fat tissue, the SVF pellet was incubated with an Fc receptor blocker (CD16/32) and a mixture of monoclonal antibodies. Cells were gated and quantified on a FACSCanto II cell analyzer, and the leukocyte population was identified on side scatter versus forward scatter plots followed by a propidium iodide (PI−)(Supplementary Fig. S5)^12^.

### Adipose tissue protein isolation, tryptic digestion, and TMT labeling

Epididymal adipose tissue from individual mice (the phenotypes of the mice subgroup is presented in Supplementary Fig. S8) was homogenized and protein extracted and digested as previously described^17^, detailed in the Supplemental Material. In brief: 100–300 mg of epididymal adipose tissue was homogenized using a tissue homogenizer and protein was extracted through sonication. Lipids were removed through centrifugation and chloroform-methanol precipitation. 110 µg protein from each sample was reduced, alkylated, and digested with LysC-Trypsin (Promega, Madison, WI) in the presence of RapiGest Surfactant (Waters, Milford, MA). Peptides were solid phase extracted using Waters Sep-Pak tC18 cartridges according to manufacturer’s instructions, dried down, and labeled with tandem-mass tag (TMT) 10-plex isobaric labels (Thermo Scientific, Rockford, IL) as previously described. Samples were then combined within their sets in 1:1:1:1:1:1:1:1:1:1 ratios based on total reporter ion intensities, determined during a normalization run, and dried down in preparation for 2D-LC-MS/MS analysis.

### LC-MS/MS & TMT data analysis

Multiplexed TMT-labeled samples were reconstituted in 5% formic acid and separated by two-dimensional reverse-phase liquid chromatography and tandem mass spectrometry data collected using an Orbitrap Fusion Tribrid instrument as previously described^17^. RAW instrument files were processed using Proteome Discoverer (PD) version 1.4.1.14 (Thermo Scientific). For each of the TMT experiments, raw files from the 9 fractions were merged and searched with the SEQUEST HT search engine with a *Mus musculus* Swiss-Prot protein database downloaded July 2015 (16,716 entries). Search results and TMT reporter ion intensities were exported as text files and processed with in-house scripts. Internal reference scaling (IRS) was used to normalize across the three TMT 10-plexes. Differential protein abundance between groups was determined by comparing the IRS-normalized total reporter ion intensities between groups using the Bioconductor package edgeR^22^. Additional data normalizations, multiple testing corrections, and calculation of false discovery rates were performed in edgeR.

### Measurement of metabolites

The procedure consisted of a solid phase extraction step, followed by a derivatization procedure using the EZ:faast amino acid analyses kit from Phenomenex (Torrance, CA). Briefly, internal standard d3-methionine was added to diluted plasma and amino acids were extracted using sorbent tips. Extracted amino acids were converted to chloroformates using reagents and instructions as described in the EZ:faast kit from the manufacturer. Derivatized amino acids were analyzed using a 4000 QTRAP hybrid/triple quadrupole linear ion trap mass spectrometer (SCIEX, Foster City, CA) with electrospray ionization (ESI) in positive mode. The mass spectrometer was interfaced to a Shimadzu (Columbia, MD) SIL-20AC XR auto-sampler followed by 2 LC-20AD XR LC pumps. Data were acquired using Analyst 1.6.2 software and analyzed using Multiquant 3.0.1 software.

### Bayesian gene regulatory networks in mouse adipose

To retrieve gene-level regulatory relations of the protein signatures detected in the study, we utilized Bayesian network models reconstructed from adipose gene expression data from multiple previously published human and mouse studies each involving genetics and gene expression data from hundreds of samples^23–26^, using an established method^23,24^. A consensus adipose network was derived from the individual networks by including only network nodes and edges that are present in networks from at least two studies.

### Key driver analysis to prioritize central regulators of gene signatures

We applied a key driver algorithm^23,25,26^ that maps the mouse protein signatures from the current study to the adipose gene regulatory network described above in order to identify the potential key regulatory genes. In this case, we defined a key driver as a gene that is connected to larger numbers of genes encoding the protein signatures, compared to the expected number of neighboring protein signatures for a randomly selected gene within a network. Statistical significance was determined using the Fisher’s exact test and multiple testing corrections were applied using the Benjamini-Hochberg FDR method^27^. In this analysis, adipose gene regulatory networks rather than protein-protein interaction networks were used, because protein-protein interaction is not required for functionally related proteins to execute their function. In contrast, regulatory networks better capture the functional relatedness and relations among genes and proteins.

### Statistical analyses

Phenotypic data were represented as means ± SDs, and ANOVA with Tukey post hoc correction was used to assess significance. Detailed statistics for each analysis are described in the corresponding sections. The mass spectrometry proteomics data have been deposited to the ProteomeXchange Consortium via the PRIDE^28^ partner repository with the dataset identifier PXD008660.

## Results

### Mice lacking GM-CSF-driven dendritic cells have comparable metabolic response to high fat and high fat plus cholesterol diets

**Figure 1:**
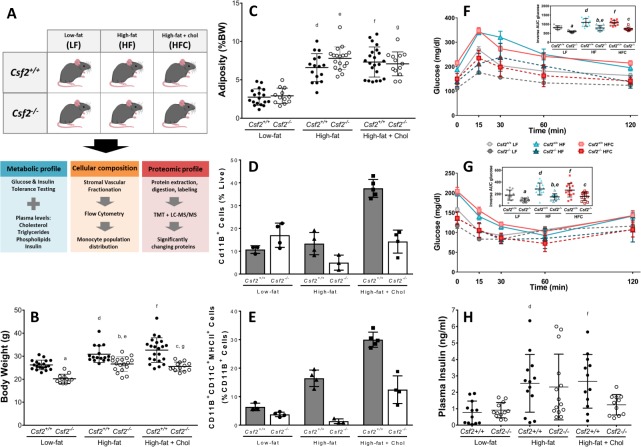
Experimental design and metabolic traits measured to study the contribution of dendritic cells to adipose tissue metabolism. (**A**) *Csf2*^+/+^ and GM-CSF deficient (*Csf2*^−/−^) male mice at 8 weeks of age were placed on low-fat (LF), high-fat (HF), or high-fat + cholesterol (HFC) diets for 8 weeks. Metabolic profile was characterized through glucose, insulin, cholesterol, triglyceride, and phospholipid measurements. Cellular composition was determined through flow cytometry on stromal vascular fractions. The whole epididymal adipose tissue proteome was analyzed for 4 mice from each experimental group through liquid-chromatography mass spectrometry using isobaric peptide labels and the key driver gene network was built from the differentially expressed protein. The following comparisons were performed for all measurements: (a) *Csf2*^+/+^ LF vs. *Csf2*^−/−^ LF, (b) *Csf2*^+/+^HF vs. *Csf2*^−/−^ HF, (c) *Csf2*^+/+^HFC vs. *Csf2*^−/−^HFC, (d) *Csf2*^+/+^LF vs. HF, (e) *Csf2*^−/−^LF vs. HF, (f) *Csf2*^+/+^LF vs. HFC, (g) *Csf2*^−/−^LF vs. HFC, (h) *Csf2*^+/+^HF vs. HFC, (i) *Csf2*^−/−^HF vs. HFC. (**B**) *Csf2*^−/−^ mice have decreased body weight compared to *Csf2*^+/+^mice on each diet at time of sacrifice. Comparisons a, b, c, d, e, f, and g, p < 0.001. (**C**) Adiposity, (epididymal + retroperitoneal adipose depots divided by body weight; details in Supplemental Fig. S1) was comparable across genotypes. For body weight and adiposity measurements; *Csf2*^+/+^ mice (low-fat (LF) n = 18, high-fat (HF) n = 16, high-fat + cholesterol (HFC) n = 22), *Csf2*^−/−^ mice (LF n = 12, HF n = 18, HFC n = 13). Comparisons d, e, f, and g, p < 0.001. Cd11B^+^ cells (**D**) and Cd11B^+^CD11C^+^MHCII^+^ cells (**E**) were quantified by flow cytometry of the stromal vascular fraction from *Csf2*^+/+^ and *Csf2*^−/−^ mice on LF, HF, and HFC diets. For flow measurements; mice were chosen around the median of the weight distribution for their group, *Csf2*^+/+^ mice (LF n = 3, HF n = 4, HFC n = 5), *Csf2*^−/−^ mice (LF n = 4, HF n = 3, HFC n = 4). Glucose (**F**) and insulin tolerance (**G**) tests were performed after a 4 h fast. For glucose tolerance tests, *Csf2*^+/+^ mice (LF n = 9, HF n = 13, HFC n = 14) and *Csf2*^−/−^ mice (LF n = 9, HF n = 9, HFC n = 8) were measured at 0, 15, 30, 60, and 120 minutes. For insulin tolerance tests, *Csf2*^+/+^ mice (LF n = 8, HF n = 13, HFC n = 14), and *Csf2*^−/−^ mice (LF n = 8, HF n = 11, HFC n = 13) were measured at 0, 15, 30, 60, and 120 minutes. Area under the curve (AUC) was calculated by normalizing to time = 0 glucose levels (inset graphs). Comparisons a, b, c, d, e, and f, p < 0.05 for both glucose tolerance and insulin tolerance tests. (**H**) Plasma insulin levels are significantly increased in *Csf2*^+/+^ mice on high-fat (HF) and high-fat + cholesterol (HFC) diets as measured by ELISA at the time of sacrifice after a 4 h fast. For *Csf2*^+/+^ mice, the number of animals were low-fat (LF) n = 12, HF n = 13, and HFC n = 12; for *Csf2*^−/−^ mice, the numbers of animals were LF n = 12, HF n = 14, and HFC n = 11. Comparisons d & f, p < 0.05. Data is presented as mean±SD for all except F and G (mean±SE). Significance was determined by ANOVA followed by Tukey’s posthoc analysis for multiple comparisons.

The experimental design is summarized in Fig. 1A. We have previously reported that mice lacking dendritic cells (*Csf2*^−/−^) exhibit increased fat mass despite reduced body weight starting early in life and throughout adulthood, when fed LF diets^12^. We assessed the weight gain and adiposity of *Csf2*^−/−^and their wild type counterparts (*Csf2*^+/+^) in response to 8 weeks of low fat (LF), high fat (HF), or high fat plus cholesterol (HFC) feeding. Despite relatively short-term diet exposure, all the mice gained weight on HF and HFC diets. However, *Csf2*^−/−^ mice exhibited ~30% significantly (P < 0.001) lower body weight in response to all diets despite comparable mouth to anus length (data not shown) (Fig. 1B). The growth curves have comparable slopes between the genotypes with *Csf2*^−/−^ being lighter from the baseline (Supplemental Fig. S1G). However, the mice had comparable weight of the epididymal adipose tissue between genotypes across diets (Supplementary Fig. S1A). Adiposity, as measured by the normalization of epididymal and retroperitoneal adipose tissue weights to total body weights, was comparable between the genotypes for all diets (Fig. 1C). Further, the adipose tissue adiponectin levels, regardless of diets, were up to 2-fold decreased in *Csf2*^−/−^ mice (Supplementary Table 1), which is in line with increased adipose tissue mass (per body weight) phenotype of these mice.

Plasma lipid profiles were measured to assess the systemic response to obesogenic diets. Plasma cholesterol, phospholipid and triglyceride levels all increased in response to HF and HFC diets in *Csf2*^+/+^ and *Csf2*^−/−^ mice (Supplementary Fig. S1D–F).

### *Csf2*^−/−^ mice have reduced dendritic cell population in adipose tissue

GM-CSF gives rise to a slew of myeloid populations, however, the tissue myeloid cell populations most affected by its loss are CD11B^+^CD11 C^−^ and CD11B^+^CD11C^+^MHCII^+^F4/80^low^ cells, the latter 3-fold higher in concentration than the former^9,12^. In our previous studies, through extensive gene expression and functional studies, we have established that CD11B^+^CD11C^+^MHCII^+^F4/80^low^ cells are GM-CSF driven dendritic cells (DCs)^9,12^. The gating strategy used in the current manuscript is identical to the one used in the previous manuscript and is presented in the Supplemental Fig. 5. Briefly, once we gate live CD45^+^CD11B^+^CD11C^+^ cells we further gate for MHCII + F4/80- cells and check the final subpopulation for other myeloid cells populations that are likely to be MHCII + with a cocktail antibody mixture that includes CD8, CD80, CD86, CD103, DEC-205. The DCs gated with our strategy are CD86, CD80, CD103, CD4, CD8a and DEC-205 negative, which eliminates other myeloid populations such as skin DCs, plasmacytoid DCs and T cells, and are functionally similar to the bone-marrow derived dendritic cells at antigen presentation^12^. Our strategy enables the identification of GM-CSF driven DCs that are conforming to the well-established dendritic cell descriptions obtained from bone-marrow derived DCs^29^. We have previously shown that as early as four-week-old and throughout a lifetime (25 week old), *Csf2*^−/−^ mice had profound reductions in CD45^+^CD11B^+^CD11C^+^ cell populations^12^. We assessed the effect of lack of GM-CSF on myeloid cell population distributions in adipose tissues of mice fed LF, HF, or HFC diets. Isolated adipose tissue stroma vascular fraction phenotypes were determined by flow cytometry. CD11B^+^ cell populations were comparable in LF, and they were reduced 20% and 50% in HF and HFC conditions in *Csf2*^−/−^ mice when compared to their wild-type littermates (Fig. 1D). We note that the lack of GMCSF also affects with a much lower degree the CD11B^-^CD11C^+^ subpopulation. We did not fully characterize these cells as they were MHCII negative.

MHCII plays an important role in antigen presentation, a major function of dendritic cells^6,30,31^. Once mature, dendritic cells contribute to antigen presentation and T cell priming^32^ and activation^30^. The CD11B^+^CD11C^+^MHCII^+^ cell population increased 2- and 3-fold with HF and HFC diets, respectively, in *Csf2*^+/+^ mice when compared to LF diet. In contrast, *Csf2*^−/−^ mice did not display a diet-dependent increase, and maintained the LF diet levels of CD11B^+^CD11C^+^MHCII^+^ cell population when fed HF and HFC diets (Fig. 1E). Altogether, the GM-CSF dependent dendritic cells were significantly reduced in adipose tissue of *Csf2*^−/−^ mice and did not increase in response to obesogenic diets. Detailed quantification of the SVF subpopulations is presented in Supplemental Fig. S2. While we show that the most affected myeloid cell population is GM-CSF driven DCs, we do not exclude the possibility that the metabolic phenotype could be due to other myeloid subpopulations that are also affected by the loss of GM-CSF.

### Lack of GM-CSF driven myeloid cells protects from obesity-induced insulin resistance

Despite comparable adiposity (Fig. 1C), *Csf2*^−/−^ mice were protected from HF diet induced glucose intolerance and insulin resistance when compared to *Csf2*^+/+^ and dietary supplementation of cholesterol had no additional effect (Fig. 1F,G). The glucose tolerance (GTT) profiles over 120 minutes, fasting glucose levels, the peak in the plasma glucose level at 15 minutes, and the glucose clearance (30–120 min) were increased for HF and HFC groups when compared to LF diet groups for both genotypes (Fig. 1F). However, these parameters were significantly decreased by at least 20% in *Csf2*^−/−^ mice (Fig. 1F). The baseline normalized area under the curves were ~50% lower in *Csf2*^−/−^ mice for all diets (Fig. 1F inset). Insulin tolerance test revealed that *Csf2*^−/−^ mice have favorable insulin sensitivity compared to *Csf2*^+/+^ mice across diets (Fig. 1G). Accordingly, following 18 weeks of HF diet, despite having ~70% increased adiposity, *Csf2*^−/−^ mice are resistant to obesity induced insulin resistance (Supplementary Fig. S7). The baseline glucose adjusted area under the curve values were ~50% lower in *Csf2*^−/−^ mice for all diets (Fig. 1G inset). Fasting plasma insulin levels were comparable between the genotypes for LF and HF groups; however, they were 50% reduced in *Csf2*^−/−^ mice fed HFC diet compared to *Csf2*^+/+^ mice (Fig. 1H). Overall the *Csf2*^−/−^ mice exhibit favorable glucose homeostasis even when challenged with obesogenic diets.

### *Csf2* and diet-dependent proteome-wide differences in adipose tissue

**Figure 2:**
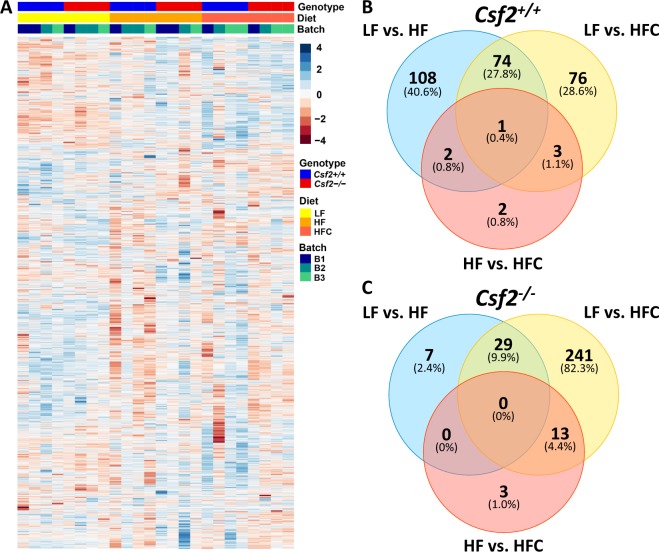
Proteomic profile of adipose tissue on low-fat, high-fat, and high-fat + cholesterol diets. 3900 proteins were measured by two-dimensional reverse-phase liquid chromatography tandem mass spectrometry and quantified by reporter ion intensities of tandem mass tags. (**A**) Sample cluster heatmap analysis was performed with Euclidean hierarchical clustering applied to measures of genotype, diet, and TMT set analysis batch. (**B**,**C**)Venn diagram of proteins with significantly different abundance (FDR < 0.05) on low-fat diet, high-fat diet, and high-fat diet + cholesterol for wildtype *Csf2*^+/+^ mice (**B**) and *Csf2*^−/−^ mice (**C**).

We assessed the proteome-wide alterations in adipose tissue in response to HF and HFC diets in *Csf2*^+/+^ and *Csf2*^−/−^ mice (Fig. 2). From a total of ~5600 proteins identified, 3900 were observed across all samples and could be quantified (Supplementary Tables S1 and S2). The protein expression levels clustered the samples according to their genotypes and the diet groups, but not by the experimental batch in which they were processed and analyzed (Fig. 2A and Supplementary Table 1). There were 185 and 152 proteins that were significantly changed (FDR < 0.05) in *Csf2*^+/+^ mice in response to HF and HFC diet when compared to LF diet, respectively (Fig. 2B). Seventy-four of these proteins were associated with complement activation (C2, C8A, C8B, C8G, C9) and lipid metabolism (ACLY, ACACA, ACSM3, ALDOA), and were shared across diets indicating that a core set of proteins participate in tissue response to high-fat diet (Fig. 2B and Supplementary Table 1).

While the *Csf2*^−/−^ mice exhibited significant (FDR < 0.05) alterations in 36 proteins in response to HF diet, addition of dietary cholesterol resulted in alteration of 283 proteins when compared to LF (Fig. 2C and Supplementary Table 1). Twenty-nine proteins associated with lipid metabolism (ACACA, ACLY, FASN, ACOX1, CYP2E1) and complement/coagulation cascade (C8B, C8G, C9) were shared between HF and HFC conditions (Fig. 2C). Adipose tissue response to dietary cholesterol led to a down regulation in complement and coagulation cascades (C3, C5, C8A, C8B, C8G, C9, HC; FGG, FGA, FGB, SERPINA3K, minimum FDR = 8.32e-09), oxidative phosphorylation (COX5B, COX5A, COX7A2, SLC25A5, UQCRH, UQCRC1, UQCRFS1, NDUFV2, NDUFA13, ATP5J, ATP8, minimum FDR = 3.76e-07), and fatty acid metabolism (ACADVL, ACACA, ACOX1, AACS, FASN, ALDH2, ACLY, AKR1B7, MGII, minimum FDR = 0.00154); with upregulation of proteins that play a role in cytoskeleton organization (CFL1, LCP1, TUBB2A, TPM4, CAP1, CRIP2, TPT1, SNCG, FHL1, SNCG TLN2, TBCB, minimum FDR = 4.98e-0.5). The profound and numerous proteome-wide changes in adipose tissue of mice lacking GM-CSF driven myeloid cells are consistent with the increased adiposity of *Csf2*^−/−^ mice in response to HFC diet. This suggests that GM-CSF driven myeloid cells alleviate in part the adipose tissue response to dietary fat and cholesterol.

### Lack of GM-CSF driven myeloid cells associate with activation of lysine and branched chain amino-acid pathways

**Figure 3:**
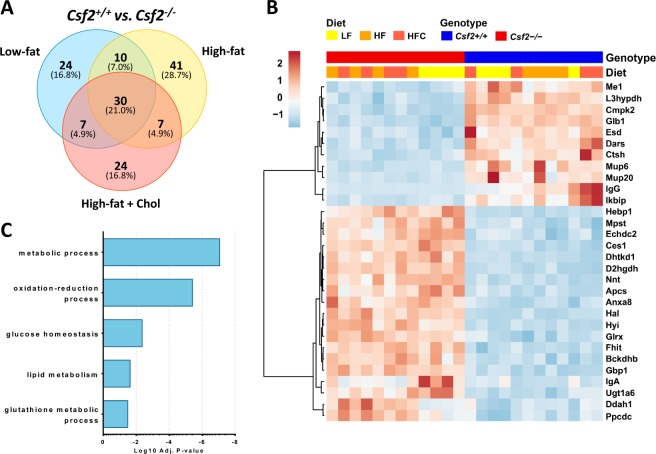
Differential protein expression in adipose tissue of mice lacking Csf2. (**A**) Significantly changing proteins (FDR < 0.05) were identified between the wildtype and Csf2^−/−^ mice, with 24 unique to low-fat diet conditions, 41 unique to high-fat diet conditions, and 24 unique to high-fat + cholesterol diet conditions, and thirty significantly changing proteins common to all diets. (**B**) Expression profiles for the thirty conserved significantly changing proteins between *Csf2*^+/+^ and *Csf2*^−/−^ across all diet types. Heatmap analysis was performed with hierarchical clustering applied to the sample and proteins. (**C**) The top five annotated biological processes that are enriched in the significantly changing proteins.

We observed 30 proteins that were significantly altered regardless of diet in *Csf2*^−/−^ mice compared to *Csf2*^+/+^ mice. GCAB, MUP6, GLB1, ME1, DARS, L3HYPDH, CMPK2, ESD, CTSH, MUP20, IGG, IKBIP were downregulated; whereas FHIT, GLRX, ANXA8, BCKDHB, NNT, HEBP1, CES1, DHTKD1, D2HGDH, APCS, HAL, HYI, MPST, ECHDC2, GBP1, IGA, UGT1A6, DDAH1, PPCDC were upregulated (Fig. 3A,B). A gene enrichment analysis using the DAVID database identified that these proteins participate in cellular metabolic process, oxido-reduction, glucose homeostasis, lipid metabolism, and glutathione metabolic process (Fig. 3C, Supplementary Table S3).

DHTKD1 participates in lysine metabolism and catalyzes the conversion of 2-oxoglutarate to succinyl-CoA and CO_2_, and impacts plasma 2-aminoadipate (2-AA) levels, which are elevated in diabetes^20^. BCKDHB (branched-chain alpha-keto dehydrogenase complex) catalyzes the overall conversion of alpha-keto acids to acyl-CoA and CO_2_, is involved in the catabolism of the branched-chain amino acids (BCAA)^33^, and strongly associates with insulin resistance, nonalcoholic fatty liver disease, nonalcoholic steatohepatitis, and hepatocellular carcinoma^17,34^. NNT (Nicotinamide nucleotide transhydrogenase) functions as a proton pump across the membrane, regulates the trans-hydrogenation between NADH and NADP, and is coupled to respiration and ATP hydrolysis. NNT has been implicated as a key mediator of increased insulin secretion, independent of changes in insulin sensitivity, in DBA/2 mouse^35^. The immunoreactivity to lysosomal enzyme GLB1 (beta galactosidase, hydrolyzes of terminal non-reducing beta-D-galactose residues in beta-D-galactosides) is increased in Type 1 diabetes in humans and rats^36^. Regardless of diet type, we have identified a differentially expressed set of mitochondrial enzymes that regulate lysine and branched-chain amino acid metabolism as in mice lacking GM-CSF.

### *Csf2*^−/−^ mice have decreased plasma 2-aminoadipate levels

**Figure 4:**
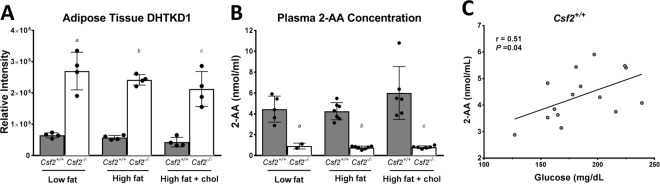
Mice lacking Csf2 have altered lysine metabolism in adipose tissue. (**A**) DHTKD1 levels are significantly increased in *Csf2*^−/−^ adipose tissue across diets. DHTKD1 measurements were performed with reporter ion intensities through two-dimensional reverse-phase liquid chromatography tandem mass spectrometry on an Orbitrap Fusion (n = 4 per experimental group). (**B**) Plasma 2-aminoadipate levels are significantly reduced in *Csf2*^−/−^ mice across diets; (*Csf2*^+/+^ low-fat (LF) n = 5, high-fat (HF) n = 7, high-fat + cholesterol (HFC) n = 6; *Csf2*^−/−^ LF n = 2, HF n = 7, HFC n = 6). Measurements were performed on derivatized amino acids with a 4000 QTRAP hybrid/triple quadrupole linear ion trap mass spectrometer. (**C**) Plasma 2-AA levels are negatively correlated with adipose DHTKD1 levels (r^2^ = 0.8652, *P* < 0.05). Data is presented as mean±SE. *D:* Plasma 2-AA levels are positively correlated with plasma glucose concentration (r^2^ = 0.5455, *P* < 0.05). Significance was determined by ANOVA followed by Tukey’s posthoc analysis for multiple comparisons and indicated as (a) *Csf2*^+/+^LF vs. *Csf2*^−/−^ LF, (b) *Csf2*^+/+^HF vs. *Csf2*^−/−^HF, (c) *Csf2*^+/+^HFC vs. *Csf2*^−/−^HFC, with p < 0.05 for each comparison.

It has been shown in mice that lower plasma 2-AA levels correlate with lower fasting glucose levels^20^. In the Framingham cohort, plasma 2-AA levels strongly associated with increased risk of developing diabetes, with the top 2-AA quartile having a 4-fold increased risk^20^. The unbiased proteomic approach used here revealed that *Csf2*^−/−^ mice had a higher abundance of DHTKD1 in their adipose tissue, which did not change with diet (Fig. 4A). Plasma 2-AA levels, measured with targeted metabolomics, were profoundly decreased in *Csf2*^−/−^ mice and did not change across diets (Fig. 4B). However, plasma 2-AA levels were slightly upregulated in *Csf2*^+/+^ mice fed HFC diet when compared to HF and LF diets (Fig. 4B). Furthermore, plasma 2-AA levels are inversely correlated with adipose tissue levels of DHTKD1 (Fig. 4C) and positively correlated with fasting plasma glucose levels (Fig. 4D).

### Key driver gene network analysis highlights that lack of GM-CSF driven myeloid cells reduced dietary sensitivity in the BCAA subnetwork

To explore the relationships among the differentially expressed proteins in response to LF, HF and HFC diets in *Csf2*^+/+^ and *Csf2*^−/−^ mice, we conducted adipose gene network analysis to identify potential key regulators of the differential proteins and extracted the subnetworks that were perturbed by the different conditions. We applied a key driver analysis (KDA; details in Materials and Methods) to the differential protein candidates using adipose tissue-specific Bayesian network models constructed from transcriptomic and genetic data sets from multiple mouse studies^37–41^, and identified up to 15 predicted putative key driver genes satisfying FDR < 0.01 in KDA for each condition (Supplementary Table S4).

**Figure 5:**
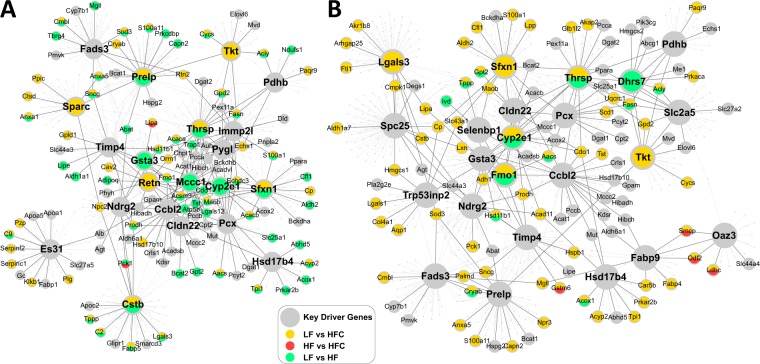
Lack of GM-CSF driven Dendritic cells impacts activation of key driver gene networks. (**A**,**B**) The diet-impacted gene networks in *Csf2*^+/+^ (**A**) and *Csf2*^−/−^ mice (**B**) are represented. Larger nodes correspond to key driver genes and nodes affected by different diets are represented by different colors. The full list of key driver genes can be found in Supplemental Table S4.

In wild-type mice (*Csf2*^+/+^), HF and HFC perturbed gene subnetworks (Fig. 5A) comprised of the top putative key drivers and their neighboring differential proteins. Key drivers shared between diets include Ctsb, Mccc1, and Sfxn1. Notably, key driver Mccc1 and its neighboring genes are mainly involved in BCAA metabolism, including Hmgcs1, Aldh2, Bcat1, Oxct1, Pccb, Pcca, Bckdhb, Mut, Hibch, Hibadh, Echs1, Dld, Bcat2, Bckdha, Auh, Aldh6a1, Acat1, and Abat.

Compared to the wildtype mice, in *Csf2*^−/−^ mice the HF and HFC perturbed subnetworks in adipose tissue were much sparser (Fig. 5B). Additionally, Mccc1 was no longer a key driver in the *Csf2*^−/−^ adipose networks. These results suggest that the lack of GM-CSF driven myeloid cells helped mitigate the perturbation in the BCAA metabolism subnetwork and reduced the sensitivity to metabolic disruptors such as HF and HFC. In contrast, several key drivers such as Thrsp, Sfxn1, Cyp2e1, and Ttk were consistent between the wildtype and knockout mice, suggesting that these genes and their subnetworks were responsive to high caloric diets independent of GM-CSF driven myeloid cells.

Due to low power, the 30 differentially expressed proteins between *Csf2*^+/+^ and *Csf2*^−/−^ mice did not populate a robust KDG network (Supplementary Fig. S4).

## Discussion

Up to 30% of obese individuals seem to be protected against obesity-related diseases including type 2 diabetes, dyslipidemia, coronary artery disease, and stroke, and have been defined as metabolically healthy. The protective molecular mechanisms that support a healthier obese state are not well understood. Although the contribution of adipose tissue to obesity-related pathologies has been widely studied from the gene expression perspective^42–44^, due to the lack of mature technologies and methods, comprehensive protein level profiling has been rare. We have used a reliable, multiplexed mass spectrometry approach^17^ coupled with phenotypic profiling and key driver gene network analysis to investigate the adipose tissue driven molecular pathways that underlie the protection from insulin resistance in obese state in a mouse model that lacks GM-CSF driven myeloid cells and that is prone to insulin sensitive obesity. The increased adiposity of the mice lacking GM-CSF develops early in life. While we did not measure the baseline adiposity in the current study, we have reported as much as 15% increase in adiposity of *Csf2*^−/−^ mice when compared with *Csf2*^+/+^ mice as early as 4 weeks old (Pamir, JBC, 2015). Further, these differences increase throughout life reaching a 2.5-fold increase at 25 weeks of age. Of note, we have used the common methods of glucose and insulin tolerance tests to assess the glucose homeostasis. However, our findings are consistent with those from the Seeley group who has used the a glucose clamp, the gold standard to measure peripheral insulin sensitivity^13^. The collective data from this and previous reports indicate that lack of GMCSF leads to a phenotype described as insulin sensitive, increased body weight and adiposity starting early in life and preserved under different diet exposures^12,13^

*In vitro*, GM-CSF treatment consistently promotes the differentiation of bone marrow progenitor cells into immature BM-DCs, and this system serves as a model for generating monocyte-derived dendritic cells that share several characteristics with their *in vivo* dendritic cell counterparts^7,8^. Specifically, BM-DCs exhibit higher levels of the cell-surface markers CD11c and MHCII and lower levels of F4/80, as compared with bone marrow-derived macrophages^9^. CD11c and MHCII are abundantly present on the dendritic cell surface^9,45^. Using these surface markers, we show that under physiological conditions, 20% of all myeloid cells in adipose tissue are GM-CSF-dependent dendritic cells^12^, and HF or HFC diet exposure does not lead to an increase in myeloid cells in *Csf2*^−/−^ mice as it does for the wild type mice. Therefore, we propose the GM-CSF driven dendritic cells are likely the most relevant myeloid population to the metabolic phenotypes we observe. These findings extend the recent data showing that adipose tissue GM-CSF driven dendritic cells are independent contributors to obesity induced insulin resistance and diabetes^2^ by exposing a weight independent link between GMCSF driven dendritic cells and insulin sensitivity. *Csf2*^−/−^ mice fed with low fat, high fat or high fat cholesterol diets exhibited protection from diet induced insulin resistance when compared with wild type mice. That said, we do not dismiss the possible contribution of other GM-CSF dependent myeloid populations, and the role of individual adipose tissue myeloid cell populations requires further characterization in future studies.

Our unbiased approach of coupling proteomics with key driver gene network analysis of the whole epididymal adipose tissue, highlights changes in mitochondrial pathways in response to obesogenic diets. These changes are more readily captured than the well documented changes in immune response. For example, branched-chain amino acid (BCAA) metabolism was profoundly impacted in response to absence of GM-CSF driven myeloid cells. This was also captured by key driver gene network analysis. Previous studies have shown that amino acid metabolism, specifically BCAA concentrations, are elevated in response to overnutrition and can affect both insulin sensitivity and secretion. Alterations in the BCAA metabolism may therefore play a role in the early pathogenesis of type 2 diabetes. Alterations in enzymes in lysine and BCAA metabolisms such as DHTKD1, BCKDHB, and the products of these pathways (e.g. 2-aminoadipic acid) have been associated with diabetes status^46^. Most of these studies are liver based and their relevance in adipose tissue is less clear. Within that perspective, our findings intriguingly link GM-CSF driven myeloid cells to the regulation of BCAA metabolism in adipose tissue for the first time.

Our findings highlight a set of 30 proteins that are responsive to the lack of GM-CSF driven myeloid cells in adipose tissue regardless of diet exposure. Once strategy in deciding which proteins to follow could have been based on the KDG analysis. Due to low power, the 30 differentially expressed proteins between *Csf2*^+/+^ and *Csf2*^−/−^ mice did not populate a robust KDG network (Supplementary Fig. S4). While pursuing all these proteins could potentially be important in describing healthy obese state, we have focused on lysine metabolism since it has recently been highlighted in two studies to be a promising player in glucose metabolism through 2-aminoadipate (2-AA)^20,47^. DHTKD1, which is ubiquitously expressed and has a high ability to catabolize lysine and tryptophan^48^. The mitochondrial levels of DHTKD1^49^, along with 2-oxoglutarate dehydrogenase regulate the 2-oxoadipate dehydrogenase activity and subsequent levels^47^. In mice and humans, SNPs associated with the Dhtkd1 gene regulate DHTKD1 protein levels, which in turn correlate with both reduced plasma 2-AA levels and plasma fasting glucose^20,47^. Further studies are required to tease out the contribution of adipose tissue lysine metabolism to plasma 2-AA levels.

**Figure 6:**
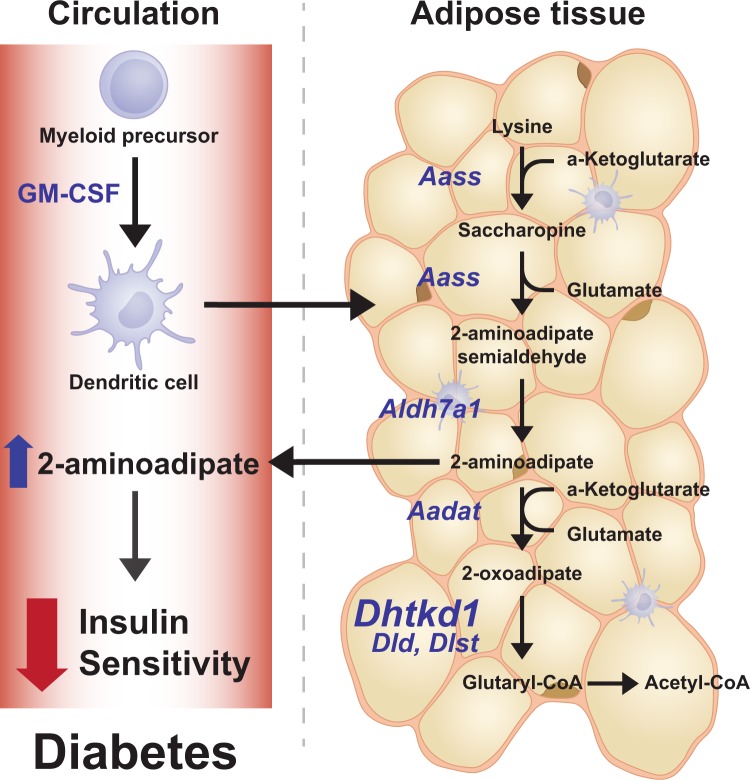
GM-CSF driven dendritic cells play a role in insulin sensitivity via the lysine metabolism involving Dhtkd1/2-AA axis. Based on our findings we propose that dendritic cells regulate adipose tissue lysine metabolism. Lack of dendritic cells leads to increased levels of DHTKD1 which in turn increases the utilization of 2-AA substrate and leads to lower plasma 2-AA levels. Plasma 2-AA levels are associated with peripheral insulin sensitivity and diabetes and are proposed to be novel biomarkers for diabetes.

Based on our unbiased systems biology approach of layering genetics, proteomics, and functional measurements we propose a model that links GM-CSF driven myeloid cells to the regulation of insulin sensitivity via lysine metabolism (Fig. 6). The global removal of GM-CSF leads to increased adiposity, profound reduction in adipose tissue GM-CSF driven myeloid cells, that is accompanied with an increase in DHTKD1 levels, lower plasma 2-AA levels, and protection from diet-induced insulin resistance. Our studies link GM-CSF driven myeloid cells to the regulation of mitochondrial activity, lysine metabolism, and levels of DHTKD1 and plasma 2-aminoadipate. The latter has been shown to be associated with the regulation of insulin sensitivity and is proposed as a biomarker for diabetes^46^ (Fig. 6). Further studies are required to fully dissect the cell types responsible for dysregulation of DHTKD1-2-AA axis in adipose tissue and in the periphery.

In an earlier report, levels of 2-AA are not well correlated with other metabolite biomarkers of diabetes, such as branched chain amino acids and aromatic amino acids, suggesting they report on a distinct pathophysiological pathway^20^. However, in our study, BCKDHB and plasma 2-AA levels were negatively and significantly correlated (Supplementary Fig. S3) suggesting that lack of GM-CSF driven myeloid cells impacts both the lysine and BCAA pathways in a coordinated manner. Further research is required to understand if other myeloid derived cells populations participate in regulation of whole adipose tissue mitochondrial metabolism.

In obese state, inflammation induces insulin resistance through a variety of molecular mechanisms^50^. Several studies with mice models have shown a disconnect between obese state and the insulin sensitivity, for example, TLR4^51^, TLR2,^52^, and MMP12^53^ knockout mice are also protected from the insulin resistance associated with diet-induced obesity, which suggests that specific obesity and insulin resistance are regulated by distinct immune pathways. Recent studies suggest that protection against development of hepatic steatosis, ectopic fat deposition, inflammation of visceral adipose tissue, and adipose tissue dysfunction, contributes to healthy obesity^54,55^. GM-CSF driven dendritic cell deficient mice have increased adiposity starting as early as 4 weeks old and throughout their life^12^ and when they are exposed to high fat and high fat diet plus cholesterol obesogenic diets for short or long term duration (Fig. 2C). Despite significantly increased adiposity they are protected from diet induced obesity related insulin resistance as shown at 8 weeks (Fig. 2G) and 18 weeks (Supplementary Fig. 6B) of HFD feeding. Within this context, our studies link GM-CSF driven myeloid cells to obesity related insulin resistance.

In summary, lack of GM-CSF driven myeloid cells protects mice from diet-induced insulin resistance but not from increased adiposity, and impacts proteome and gene network signatures associated with the BCAA and lysine metabolism. Furthermore, plasma levels of 2-AA, the key metabolite of the lysine metabolism and an emerging diabetes marker, were reduced in mice lacking GM-CSF driven dendritic cells. Our results suggest that GM-CSF driven myeloid cells participate in the adipose tissue mitochondrial metabolism which in turn regulates the peripheral insulin sensitivity via DHTKD1/2-AA axis (Fig. 6).

## Electronic supplementary material

Supplementary Material

Supplemental Table
