# Supplementary material for: GM-CSF driven myeloid cells in adipose tissue link weight gain and insulin resistance via formation of 2-aminoadipate
Source: Sci Rep. 2018 Jul 31;8:11485. doi: 10.1038/s41598-018-29250-8 (PMC6068153; doi:10.1038/s41598-018-29250-8)
Supplement: Supplementary file 1 — Supplementary Material [file 41598_2018_29250_MOESM1_ESM.docx]

**GM-CSF driven myeloid cells in adipose tissue link weight gain and insulin resistance via formation of 2-aminoadipoate**

Deanna L. Plubell^1^, Alexandra M. Fenton^1^, Phillip A. Wilmarth^2^, Paige Bergstrom^1^, Yuqi Zhao^3^, Jessica Minnier^1^, Jay W. Heinecke^4^, Xia Yang^3^, Nathalie Pamir^1^

**SUPPLEMENTAL DATA**

**Mice.**

Wild-type *Csf2^+/+^* male mice (Jackson Labs, #000664) and knock-out *Csf2^-/^*^-^ male mice (a kind gift from Randy Seeley) (both in a C57BL/6 background) were housed within a specific pathogen-free barrier facility in a temperature-controlled room (22°C) with a 12-h light/dark cycle, and given free access to food and water (3−5 mice per cage). Because the *Csf2^-/-^* mice were bred >12 generations to C57Bl/6 mice, we used Jackson delivered C57BL/6 mice as controls. At 8 weeks of age, the mice were fed either a low-fat (4%) regular chow diet (Wayne Rodent BLOX 8604; Harlan Teklad Laboratory, Madison, WI), a high-fat diet (HF diet; 60% fat calories, D12492 Research Diets) or a HF diet supplemented with cholesterol (HF+C diet; 0.15% cholesterol w/w, 60% fat calories, F4997 Bio-Serv) and were analyzed at 16 weeks age. Before necropsy, mice were fasted for 4 h in the morning, bled from the retro-orbital sinus into tubes containing 1 mM EDTA, and euthanized by isofluorane inhalation. Epididymal adipose depots were collected, weighed, snap frozen in liquid nitrogen, and stored at −80°C until analysis. A subset of whole epididymal adipose depots were isolated and digested immediately for flow cytometry experiments.

**Adipose tissue fractionation**

Under sterile conditions, adipose tissue was extracted and separated into stromal vascular cell and adipocyte fractions. Minced tissue in digestion buffer (Dulbecco’s PBS supplemented by calcium and magnesium, Thermo Scientific, Rockford IL) was incubated with 2 mg/ml type I collagenase (Worthington Biochemical, Lakewood, NJ) for 45 min at 37 °C on an orbital shaker, filtered through 250 µm nylon mesh, and centrifuged at 500 x *g* for 5 min. The pellet was resuspended in erythrocyte lysis buffer (Cell Signaling, Danvers, MA), incubated at room temperature for 5 min, and then filtered through a 70 µm nylon mesh and washed by centrifugation as above.

**Antibodies for flow cytometry**.

Propidium iodide was used to eliminate dead cells from the analysis. Antibodies were purchased from eBioscience: Cd45 (48-0451), Cd11b (25-0112), Cd11c (12-0114), F4/80 (11-24801), MHCII (11-5980), PI (00-6990), and Fcblock (14-0161).

**Protein extraction and digestion**

100 – 300 mg of epididymal adipose tissue from individual mice was homogenized on ice in 1 ml homogenization buffer (150mM NaCl, 50 mM HEPES pH 8.5, 1x GBiosciences ProteaseArrest) using a polytron tissue homogenizer. Homogenized samples were spun at 10,000 g for 10 min at 4°C and the top lipid layer removed. Supernatant and pellet were lysed by addition of SDS to a final concentration of 2.5% and sonicated three times at 2 watts for five seconds each with 30 second rests between. Chloroform-methanol precipitation was performed to further eliminate lipids. In brief, four parts methanol, two parts chloroform, and three parts water were added to each sample, mixed, and spun down. The top fraction was removed and the protein layer was washed four times with 100% methanol. Proteins were dried by vacuum centrifugation and stored at -80°C. Protein samples were reconstituted in 50 mM HEPES and protein concentration was determined by the bicinchoninic acid (BCA) assay with a BSA standard (Thermo Scientific).

Epididymal adipose protein samples were reconstituted in 50 mM HEPES with brief sonication to aid in protein solubilization, and protein concentration was determined using the Pierce bicinchoninic acid (BCA) protein assay with a BSA standard (Thermo Fisher Scientific). The following steps were carried out with 110 μg protein of each sample in 0.1% Rapigest (Waters, Milford, MA). Disulfide bonds were reduced with 5 mM tris(2-carboxyethyl) phosphine (TCEP) for 30 min at 37°C. Cysteines were alkylated with 15mM iodoacetamide for 30 min at room temperature in the dark. Excess iodoacetamide was quenched with 5 mM DTT for 15 min at room temperature in the dark. Protein was digested with a mixture of LysC and Trypsin (Cat # V5071, Promega, Madison, WI) at a 1:100 w/w protease:protein ratio for 3 h at 37°C, then at a 1:50 w/w ratio overnight at 37°C. Digestion was terminated by the addition of trifluoroacetic acid to 0.5%. Particulates were then removed by spinning at 12,000 g for 15 min, and peptides were solid phase extracted using Waters Sep-Pak tC18 cartridges according to manufacturer’s instructions, dried down, and stored at -80°C.

**TMT labeling**

Peptides were reconstituted in 100 mM triethylammonium bicarbonate (TEAB) and their concentration determined by the BCA assay described above. A pooled sample for internal reference scaling (IRS) normalization between runs was prepared by combining an equal ratio of peptide from each individual sample. Samples were randomly distributed between 3 sets of TMT 10-plexes, with 2 tags from each set reserved for a pooled internal standard for IRS normalization. 25 µg from each sample was labeled. Equal volumes from each sample within a set were combined and analyzed through LC-MS/MS using an Orbitrap Fusion (Thermo Scientific) in order to determine labelling efficiency.

**LC-MS/MS**

Multiplexed TMT-labeled samples were reconstituted in 5% formic acid and separated by two-dimensional reverse-phase liquid chromatography using a Dionex NCS-3500RS UltiMate RSLCnano UPLC system. A 20 μl sample (40 μg) was injected onto a NanoEase 5 μm XBridge BEH130 C18 300 μm x 50 mm column (Waters) at 3 μl/min in a mobile phase containing 10 mM ammonium formate (pH 9). Peptides were eluted by sequential injection of 20 μl volumes of 14, 20, 22, 24, 26, 28, 30, 40, and 90% ACN in 10 mM ammonium formate (pH 9) at 3 μl/min flow rate. Eluted peptides were diluted with mobile phase containing 0.1% formic acid at 24 μl/min flow rate and delivered to an Acclaim PepMap 100 μm x 2 cm NanoViper C18, 5 μm trap on a switching valve. After 10 min of loading, the trap column was switched on-line to a PepMap RSLC C18, 2 μm, 75 μm x 25 cm EasySpray column (Thermo Scientific). Peptides were then separated at low pH in the 2nd dimension using a 7.5–30% ACN gradient over 90 min in mobile phase containing 0.1% formic acid at 300 nl/min flow rate. Each 2nd dimension LC run required 2 hours for separation and re-equilibration, so each 2D LC-MS/MS method required 18 hours for completion. Tandem mass spectrometry data was collected using an Orbitrap Fusion Tribrid instrument configured with an EasySpray NanoSource (Thermo Scientific). Survey scans were performed in the Orbitrap mass analyzer (resolution = 120,000), and data-dependent MS2 scans performed in the linear ion trap using collision-induced dissociation (normalized collision energy = 35) following isolation with the instrument’s quadrupole. Reporter ion detection was performed in the Orbitrap mass analyzer (resolution = 60,000) using MS3 scans following synchronous precursor isolation of the top 10 ions in the linear ion trap, and higher-energy collisional dissociation in the ion-routing multipole (normalized collision energy = 65).

**MS data processing**

RAW instrument files were processed using Proteome Discoverer (PD) version 1.4.1.14 (Thermo Scientific). For each of the TMT experiments, raw files from the 9 fractions were merged and searched with the SEQUEST HT search engine with a Mus musculus Swiss-Prot protein database downloaded July 2015 (16,716 entries). Searches were configured with static modifications for the TMT reagents (+229.163 Da) on lysines and N-termini, carbamidomethyl (+57.021 Da) on cysteines, dynamic modifications for oxidation of methionine residues (+15.9949 Da), parent ion tolerance of 1.25 Da, fragment mass tolerance of 1.0005 Da, monoisotopic masses, and trypsin cleavage (max 2 missed cleavages). Searches used a reversed sequence decoy strategy to control peptide false discovery and identifications were validated by Percolator software. Only peptides with q scores < 0.05 were accepted, and at least one unique peptide was required for matching a protein entry for its identification. Default protein identification criteria was used (protein false discovery analysis was not reported by PD 1.4).

Search results and TMT reporter ion intensities were exported as text files and processed with in-house scripts. A median reporter ion intensity peak height cutoff of 150 was used, and all reporter ion intensities for unique peptides matched to each respective protein were summed to create total protein intensities. We employed two normalization procedures to handle the 30-plex experiment (3 TMT experiments with 10 channels each). The first was applied within each 10-plex experiment. The grand total reporter ion intensity for each channel was multiplied by global scaling factors to adjust its total intensity to the average total intensity across the 10 channels. Common, pooled internal standards were used to normalize reporter ion intensities of proteins between different TMT experiments. The duplicate summed reporter ion measurements for each protein in the common pool channels within each TMT experiment were averaged and used to create reference values for each protein. The three reference values for each protein in each TMT experiment were then averaged (geometric mean), and scaling factors calculated for each protein to adjust its reference value to the geometric mean value. These scaling factors were then used to adjust the summed reporter ion intensities for each protein in the remaining 8 experimental samples in each TMT experiment. Quantifiable proteins were restricted to proteins identified in all three experiments and should have a low protein false discovery rate.

**Metabolite measurements & analysis**

Briefly, internal standard (d3-methionine) was added to diluted plasma (10 µL plasma plus 90 µL of phosphate buffered saline, PBS) and amino acids were extracted using sorbent tips. Extracted amino acids were converted to chloroformates using reagents and instructions as described in the EZ:faast kit from the manufacturer. Standards containing from 0.1 to 20 nmol/mL were prepared in 100 µL PBS at the same time {Fonteh:2007kx}. Derivatized amino acids were analyzed using a 4000 QTRAP hybrid/triple quadrupole linear ion trap mass spectrometer (SCIEX, Foster City, CA) with electrospray ionization (ESI) in positive mode. The mass spectrometer was interfaced to a Shimadzu (Columbia, MD) SIL-20AC XR auto-sampler followed by 2 LC-20AD XR LC pumps. The instrument was operated with the following settings: source voltage 4500 kV, GS1 50, GS2 50, CUR 20, TEM 350 and CAD gas medium. The multiple reaction monitoring (MRM) transitions were as follows: α-amino adipic acid, m/z 332→244 (quantifier ions) m/z 332→272 (qualifier ions); d3-methionine, m/z 281→193 and m/z 281→142. All transitions were obtained with a DP 56, CE 25, CXP 10 and each was monitored with a 50 ms dwell time. The gradient mobile phase was delivered at a flow rate of 0.25 ml/min and consisted of two solvents, 10 mM ammonium formate (solvent A) and 10 mM ammonium formate in methanol (solvent B). Initial concentration of B was 70%, which was held for 0.1 min followed by an increase to 98% B by 6 minutes, held at 98% to 8 min decreasing to 70% again over 0.1 minutes, followed by re-equilibration for 5 min. The column was a EZ:faast AAA-MS column, 250x2 mm, maintained at 40°C using a Shimadzu CTO-20AC column oven. Data were acquired using Analyst 1.6.2 software and analyzed using Multiquant 3.0.1 software.

Supplementary Table 1: The link to the table is below.

https://www.dropbox.com/s/n1o3lwzoojbek54/Supplemental_Table_1_Sept.xlsx?dl=0

Supplementary Table S1. **Compiled proteomics data and analysis.** Reporter ion intensity data acquired from each TMT 10-plex was compiled, normalized, and analyzed. It is presented as a separate excel file due to its complexity and size.

Supplementary Table S2. **Results from each TMT experiment database search.** For each TMT 10-plex (A, B, or C), the raw files from the 9 fractions were merged and searched with the SEQUEST HT search engine with a Mus musculus Swiss-Prot protein database. PSMs denotes peptide-spectrum-matches, q-value is from Percolator where relaxed is 0.05 and strict is 0.01, the intensity threshold for TMT ions was median intensity in excess of 150.

Supplementary Table S3. **Enrichment pathway analysis by DAVID software**. The first column is the Gene Ontology (GO) term accession, the next column is the representative GO title, the Count column is the observed frequency of the term, the Expected column is predicted frequency for the number of observed genes, the Fold Enrichment column is how much greater the observed frequency was compared to predicted frequency, and the P-value is the probability that the observed frequency occurred by chance, with the Bonferroni Adj. P-value being adjusted for multiple testing.

| **Term** | **Representative term** | **Count** | **Fold Enrichment** | **P-value** | **Bonferroni Adj. P-value** |
| --- | --- | --- | --- | --- | --- |
| GO:0008152 | Metabolic process | 20 | 6.73 | 1.00E-10 | 8.71E-08 |
| GO:0055114 | Oxidation-reduction process | 21 | 4.84 | 8.94E-09 | 3.89E-06 |
| GO:0042593 | Glucose homeostasis | 6 | 32.47 | 1.50E-05 | 4.33E-03 |
| GO:0045834 | Lipid metabolism | 4 | 41.57 | 1.08E-04 | 2.32E-02 |
| GO:0006749 | glutathione metabolic process | 5 | 15.91 | 2.64E-04 | 3.23E-02 |

Supplementary Table S4. **Key Driver Gene Analysis.**


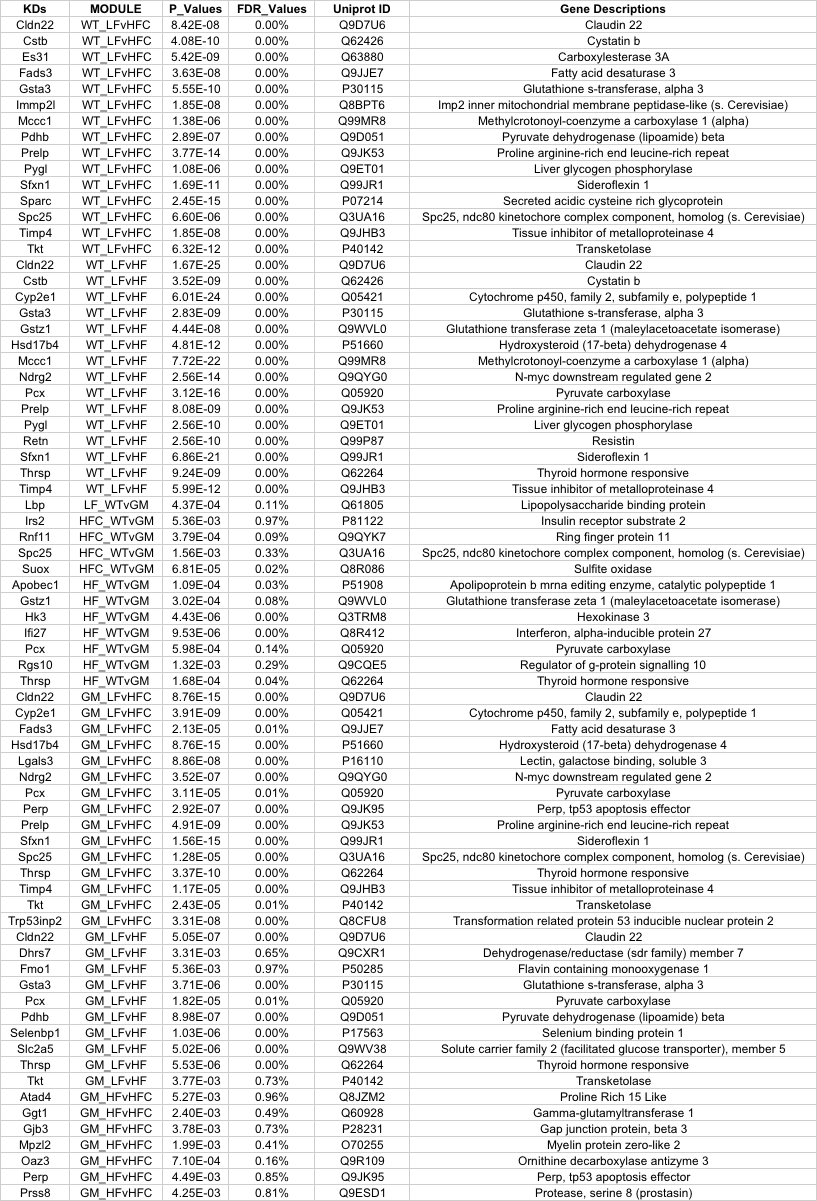


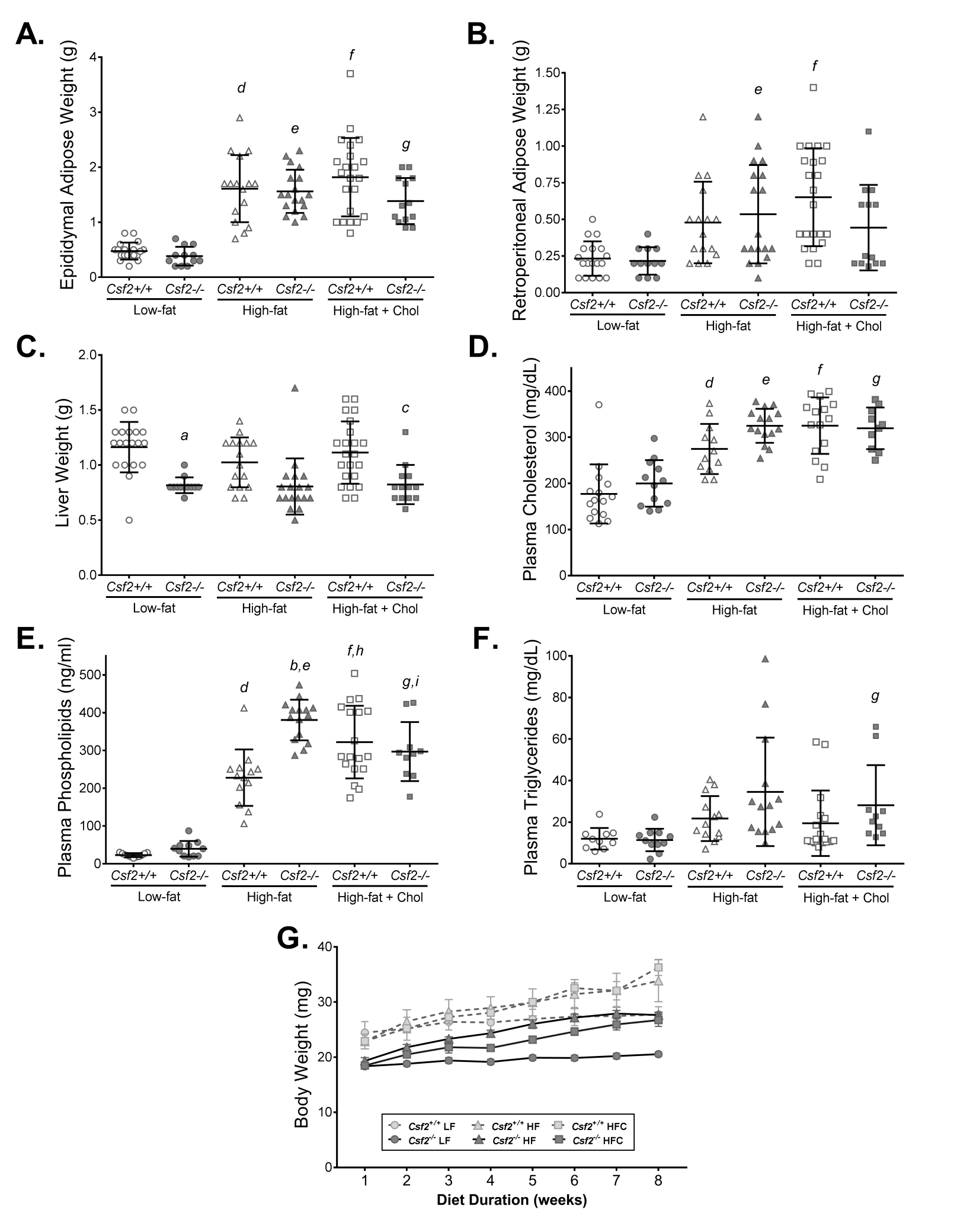


Supplementary Figure S1. **Metabolic measurements for wildtype (Csf2+/+) and GM-CSF deficient (Csf2-/-) mice on low-fat (LF), high-fat (HF), and high-fat + cholesterol (HFC) diets.** *A:* Mice on HF and HFC diets have increased epididymal adipose tissue weight compared to mice on LF for both *Csf2^+/+^* (LF n=19, HF n=16, HFC n=22) and *Csf2^-/-^* (LF n=12, HF n=18, HFC n=13). *B:* Mice on HF and HFC diets have increased epididymal adipose tissue weight distribution compared to mice on LF for both *Csf2^+/+^* (LF n=19, HF n=16, HFC n=22) and *Csf2^-/-^* (LF n=12, HF n=18, HFC n=13).*C:* Liver tissue weight is lower in *Csf2^-/-^* mice (LF n=12, HF n=18, HFC n=13), compared to *Csf2^+/+^* mice (LF n=19, HF n=16, HFC n=22). *D:* Mice on HF and HFC diets have increased plasma cholesterol levels compared to mice on LF for both *Csf2^+/+^* (LF n=15, HF n=12, HFC n=18) and *Csf2^-/-^* (LF n=12, HF n=15, HFC n=10). *E:* Mice on HF and HFC diets have increased plasma phospholipids levels compared to mice on LF for both *Csf2^+/+^* (LF n=11, HF n=13, HFC n=18) and *Csf2^-/-^* (LF n=11, HF n=15, HFC n=10). *F:* Plasma triglyceride measurements of *Csf2^+/+^* (LF n=10, HF n=13, HFC n=17) and *Csf2^-/-^* (LF n=11, HF n=14, HFC n=10). *G:* The body weight profiles increase over an 8 week diet duration, with the weights of *Csf2^-/-^* (LF n=19, HF n=9, HFC n=9) lower than corresponding *Csf2^+/+^* (LF n=18, HF n=13, HFC n=19). All error bars are standard deviation. Significance was determined by ANOVA followed by Tukey’s posthoc analysis for multiple comparisons and indicated as (a) *Csf2^+/+^­­* LF vs. *Csf2^-/-^* LF, (b) *Csf2^+/+­­^* HF vs. *Csf2^-/-^* HF, (c) *Csf2^+/+­­^* HFC vs. *Csf2^-/-^* HFC, (d) *Csf2^+/+­­^* LF vs. HF, (e) *Csf2^-/-­­^* LF vs. HF, (f) *Csf2^+/+­­^* LF vs. HFC, (g) *Csf2^-/-­­^* LF vs. HFC, (h) *Csf2^+/+­­^* HF vs. HFC, (i) *Csf2^-/-­­^* HF vs. HFC.

Supplementary Figure S2. **Additional flow cytometry data.** SVF isolated from Csf2-/- and Csf2+/+ mice fed LF (A and D), HF (B and E), and HFC (C and F) for 8 weeks. The SVF is isolated and the cells are labeled with CD11B, CD11 C, and F480 antibodies. Live (PI-) and F4/80 low cells are sorted according to their surface expression of CD11B and CD11C (A-C). CD11B positive cells are further sorted according to their expression of CD11C and MHCII (B-F). N=4, ****P*<0.0001, ***P*<0.001, **P*<0.05.


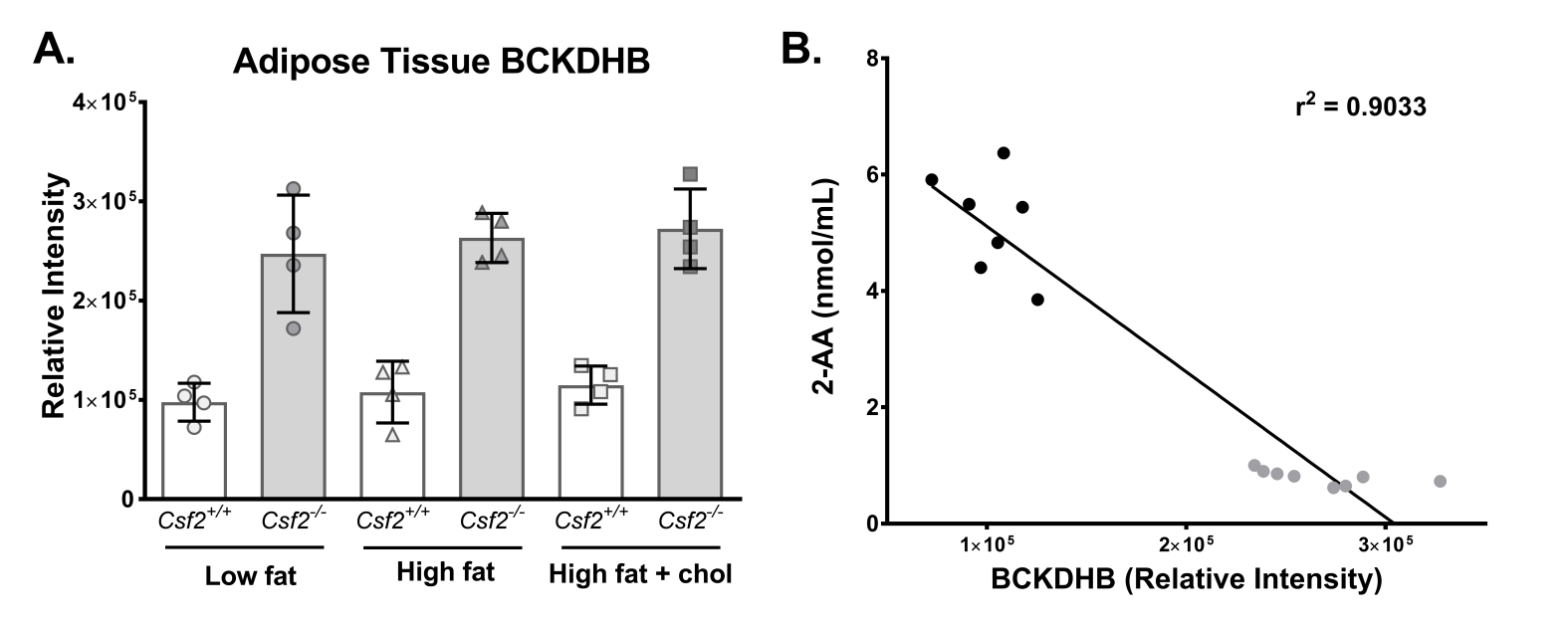


*c*

*b*

*a*

Supplementary Figure S3. **Adipose tissue BCKDHB and plasma 2-AA levels**. *A:* BCKDHB levels are significantly increased in *Csf2^-/-^* adipose tissue across diets. BCKDHB measurements were performed with reporter ion intensities through two-dimensional reverse-phase liquid chromatography tandem mass spectrometry on an Orbitrap Fusion (n=4 per experimental group). *B:* Plasma 2-AA levels are negatively correlated with adipose BCKDHB levels (r^2^ = 0.9033). All error bars are standard error of the mean. Significance was determined by ANOVA followed by Tukey’s posthoc analysis for multiple comparisons and indicated as (a) *Csf2^+/+^­­* LF vs. *Csf2^-/-^* LF, (b) *Csf2^+/+­­^* HF vs. *Csf2^-/-^* HF, (c) *Csf2^+/+­­^* HFC vs. *Csf2^-/-^* HFC.


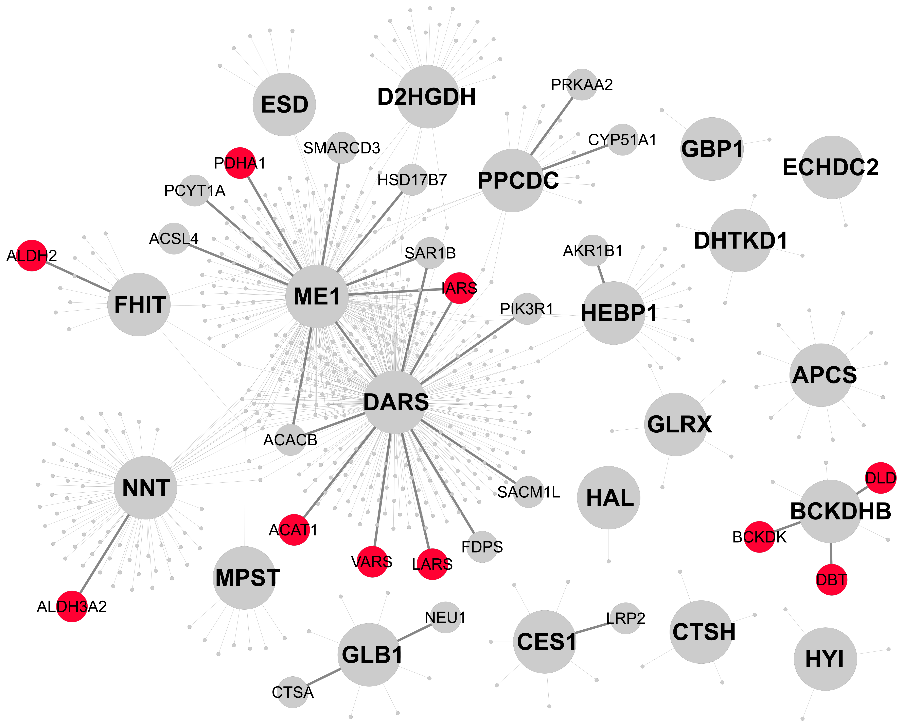


Supplementary Figure S4. **Network analysis of significantly changing protein due to genotype.** The PPI InWeb subnetwork of the 30 significantly changing proteins between *Csf2^+/+^* and *Csf2^-/-^* mice on all diets, with key drivers in red.

Supplementary Figure S5. **The FACS sorting strategy.** Following a side and front scatter, live cells are gated by being Propidium iodide negative. CD11B and CD11C positive and negative populations are gated for MHCII presence and for F4/80 presence. CD11B+CD11C+ MHCII+F4/80low gate is further validated to identify these cells as CD80-CD86-CD103-CD4-CD8a-Dec205 negative^12^.


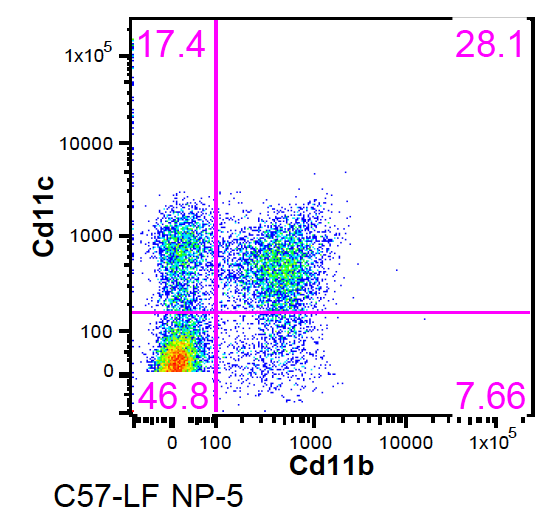

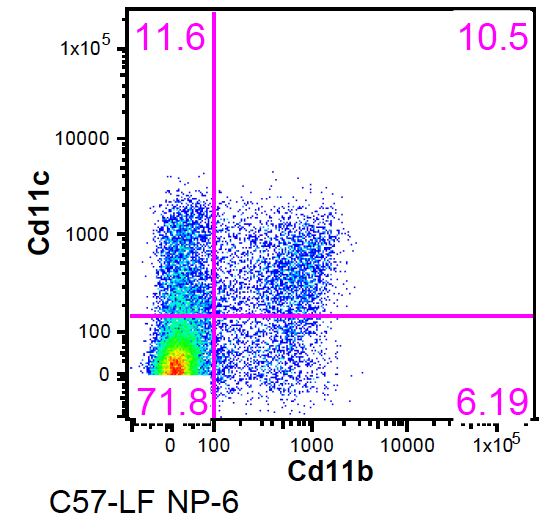

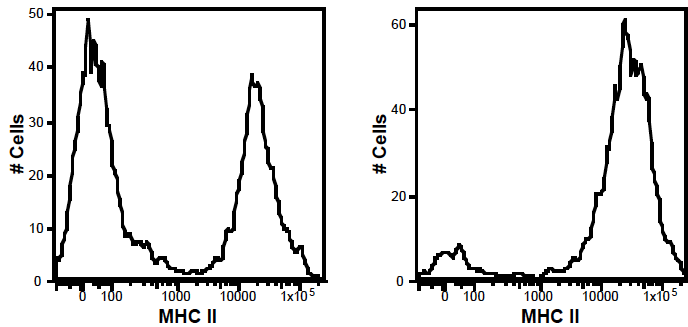

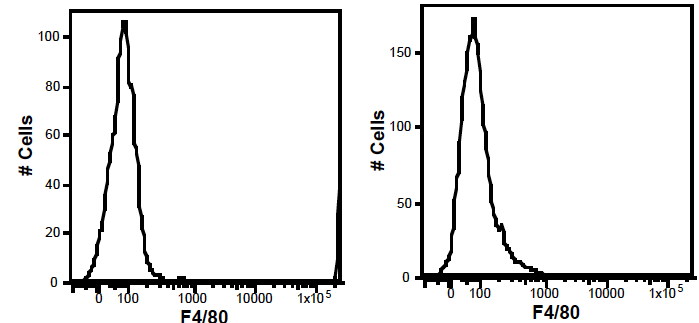


Supplementary Figure S6. **The representative FACS gates.** Flow sorting strategies for the adipose tissue SVF isolated from Csf2^+/+^ mice fed LF for 8 weeks.

.


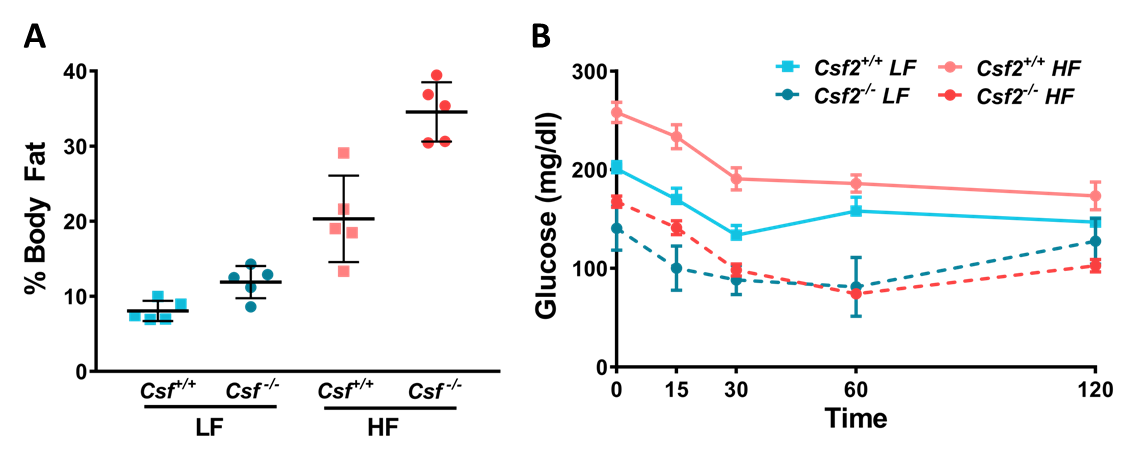


Supplementary Figure S7. **Effect of longer diet duration on adiposity and insulin sensitivity**. *A:*  Body Fat percentage as measured by the normalization of epididymal adipose weights to total body weight (n=5 for all groups). *B:* Insulin Tolerance Test for mice fed 18 weeks of HFD. Longer diet exposure introduced an overt insulin resistance in *Csf2^+/+^* (LF n=5, HF n=11) but not in *Csf2^-/-^* mice (LF n=13, HF n=15).


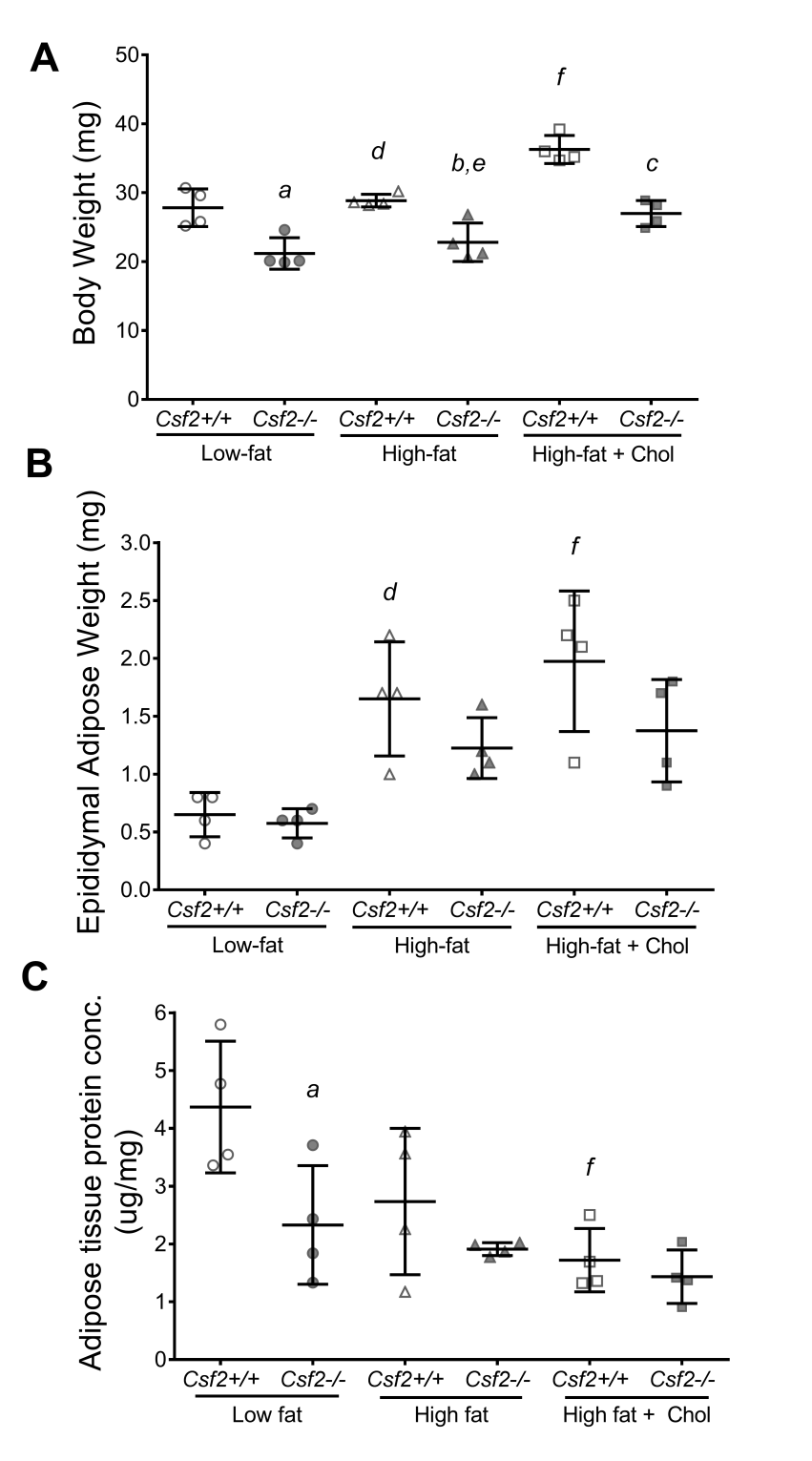


Supplementary figure S8. **Measurements for mice used for adipose tissue proteomic analysis**. *A:* The body weight for the subset of mice used for proteomics analysis follows the larger population trends. *B:* The total epididymal adipose tissue depot weight for the subset of mice used for proteomics analysis generally follow the larger population trend, but with some decrease in HF *Csf2^-/-^*. *C:* Mice on HF and HFC diets have lower protein concentrations in the epididymal adipose tissue for *Csf2^+/+^*, but not significantly in *Csf2^-/-^*. All error bars are standard deviation, n=4 for all groups. Significance was determined by ANOVA followed by Tukey’s posthoc analysis for multiple comparisons and indicated as (a) *Csf2^+/+^­­* LF vs. *Csf2^-/-^* LF, (b) *Csf2^+/+­­^* HF vs. *Csf2^-/-^* HF, (c) *Csf2^+/+­­^* HFC vs. *Csf2^-/-^* HFC, (d) *Csf2^+/+­­^* LF vs. HF, (e) *Csf2^-/-­­^* LF vs. HF, (f) *Csf2^+/+­­^* LF vs. HFC, (g) *Csf2^-/-­­^* LF vs. HFC, (h) *Csf2^+/+­­^* HF vs. HFC, (i) *Csf2^-/-­­^* HF vs. HFC.
